# A 55-year-old craftsman with dyspnea and clubbing: a case report

**DOI:** 10.4076/1757-1626-2-8579

**Published:** 2009-09-09

**Authors:** Stefan Scheidl, Gabor Kovacs, Elvira Stacher, Helmut Popper, Horst Olschewski

**Affiliations:** 1 Division of Pulmonology, Department of Internal Medicine, Medical University GrazAuenbruggerplatz 20, 8036 GrazAustria; 2 Institute of Pathology, Medical University GrazAuenbruggerplatz 25, 8036 GrazAustria

## Abstract

**Introduction:**

Clubbing is very uncommon in respiratory bronchiolitis-associated interstitial lung disease, and primarily raises the suspicion of idiopathic pulmonary fibrosis in a patient presenting with diffuse parenchymal lung disease. If idiopathic pulmonary fibrosis can be excluded, clubbing should raise the suspicion of an occult tumour.

**Case presentation:**

We describe a heavy smoker presenting with dyspnea and severe clubbing. Surgical lung biopsy revealed the histologic diagnosis of respiratory bronchiolitis-associated interstitial lung disease. Respiratory bronchiolitis-associated interstitial lung disease is a distinct clinicopathologic entity within idiopathic interstitial pneumonia patients described almost exclusively in cigarette smokers. The disease is associated with a good prognosis and mild symptoms but not with clubbing. After smoking cessation the radiologic findings of interstitial lung disease improved in parallel with improvement in lung function and gas exchange. However, a central squamous cell carcinoma was detected in the follow-up.

**Conclusion:**

In this case, clubbing was most probably caused by the occult tumor rather than by respiratory bronchiolitis-associated interstitial lung disease.

## Introduction

Cigarette smoking is a well-recognized cause of a variety of lung diseases, including interstitial lung disease. Clubbing in itself is very uncommon in respiratory bronchiolitis-associated interstitial lung disease (RB-ILD), and primarily raises the suspicion of idiopathic pulmonary fibrosis (IPF) in a patient presenting with diffuse parenchymal lung disease (ILD). If IPF is excluded histologically, clubbing should raise the suspicion of an occult tumour such as non small cell lung cancer.

## Case presentation

**Figure 1.**
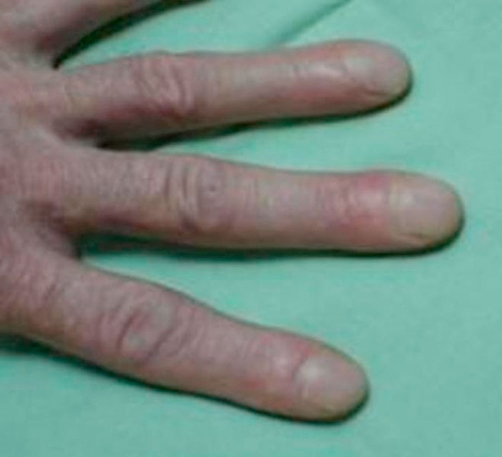
Digital clubbing has rarely been reported in RB-ILD, but is frequently associated with tumorous diseases and IPF.

**Figure 2.**
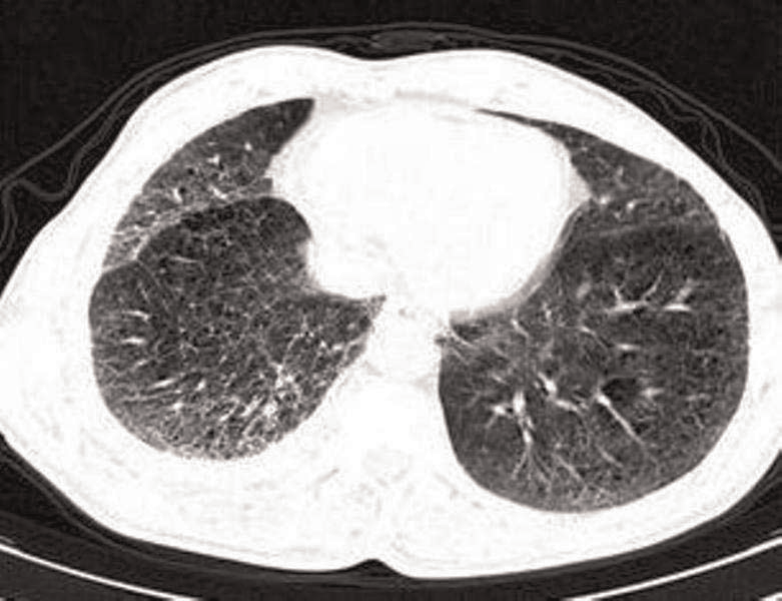
HR-CT showing diffuse ground glass opacities predominantly involving the lung basis. This aspect being consistent with the clinical finding of fine inspiratory basal crackles at auscultation.

**Figure 3.**
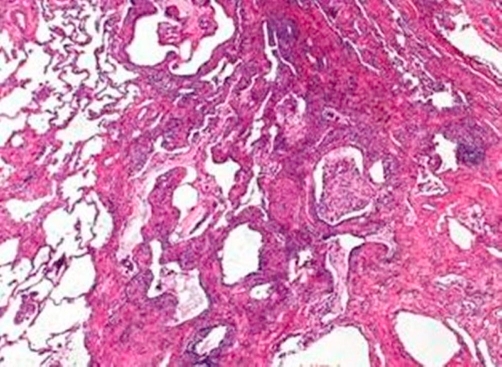
Pigment laden intraalveolar macrophages in patchy aggregates predominantly in the lumen of the bronchioles extending into the alveolar space at multiple foci.

**Figure 4.**
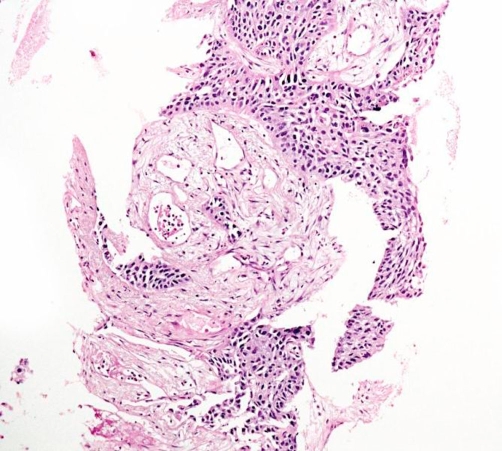
40x: The fragmented biopsy consists entirely of malignant tumour tissue and tumour stroma. No peripheral pulmonary tissue is seen.

A 55-year-old Caucasian who had been a heavy smoker (up to 40 cigarettes per day) over 37 years reported to our outpatient clinic because of increasing dyspnea and persistent cough. He had been working as craftsman for 35 years with a history of exposure to cement dust, but not to birds or other animals, fungi or other organic fine particles. He did not report weight loss, sweat or fever. On examination he had normal vital signs and was comfortable at rest. Extremities showed considerable clubbing of fingers (Figure 1) and toes. Chest examination revealed fine inspiratory basal crackles. Laboratory testing revealed a normal red and white blood count (Hb 15 g/dL; WBC 9.7 × 10^3^/µL), and a normal differential WBC (0% Eosinophils) and platelet count (331 × 10^3^/µL). Routine clinical chemistry was within the normal range, IgG, rheumatoid factor, antinuclear antibodies and anticytoplasmatic antibodies were all negative, IgE, ACE, NT-proBNP and α-1-antitrypsin levels were within the normal range. A room air arterial blood gas analysis showed pH 7.4; PaO_2_ 80 mm Hg; PaCO_2_ 40 mm Hg; O_2_Sat 96 %; HCO_3_ 24 mEq/L; resting alveolar-arterial oxygen tension gradient 21.5 mm Hg. The echocardiogram was normal. Chest radiograph showed reticulonodular infiltrates in both lung bases. HRCT showed diffuse ground glass opacities predominantly involving the lung bases, and subpleural fibrosis (Figure 2). There was one central irregular opacity of 1.5 cm in diameter which was planned for biopsy. Pulmonary function test (PFT) showed: FEV1 2.26 L (65% pred); FVC 2.42 L (56% pred); FEV1/FVC 94%; a total lung capacity (TLC) of 81% predicted, and residual volume of 124%; diffusing capacity of the lung for carbon monoxide (D_LCO_) 4 mmol/min/kPa (45% pred), consistent with a mild to moderate obstruction, a moderate restriction, and a moderate to severe diffusion abnormality. By means of video assisted thoracoscopy, peripheral wedge resections were taken from two lobes. These showed abundant pigment-laden intraalveolar macrophages in patchy aggregates, predominantly in the lumen of the bronchioles but extending into the alveolar space at multiple foci, following a peribronchial distribution (Figure 3). There was thickening of the bronchiolar walls by smooth muscle hypertrophy and mild edema, but no evidence of alveolar interstitial fibrosis, and no evidence of lung cancer. These histologic findings, together with the clinical features were interpreted as RB-ILD. A biopsy of the central opacity showed fibrotic organisation without signs of malignancy. The patient was advised to quit smoking. On the day before surgical procedure the patient stopped smoking and did not start again. No other measures were taken after the lung biopsy. After 10 months of non-smoking, his symptoms had improved and he was able to perform his daily activities without dyspnea. Repeated PFT´s and radiography showed slight improvement. On the CT control there was some improvement in the interstitial changes, however, the central nodule had grown to 1.7 cm in diameter, and a CT-guided needle biopsy revealed a squamous cell carcinoma (Figure 4), which, at that point, was not operable. The patient was staged and chemotherapy was applied for the following 18 months, until the patient died, 42 months after initial presentation.

## Discussion

Cigarette smoking is a well-recognized cause of a variety of lung diseases, including interstitial lung disease [1]. Although RB-ILD is regarded as one manifestation of the parenchymal response to cigarette smoke inhalation in both animals and humans [2,3], the pathologic mechanisms are poorly understood. RB-ILD is associated with interstitial edema and intraalveolar macrophage accumulation, a key feature of interstitial inflammation [4]. Most patients with RB-ILD are either asymptomatic or describe only mild symptoms, they are typically young (mean age 36 years), with an average of 30 to 40 years of smoking history [5]. When present, symptoms include minimally productive cough and dyspnea. Physical signs include late inspiratory crackles, but no velcro rales. RB-ILD has a much better outcome than other forms of idiopathic interstitial pneumonia [6], and it is important to establish this diagnosis because of its significantly better prognosis, and to avoid unnecessary treatment with potentially toxic medications. Clubbing in itself is very uncommon in RB-ILD [4,7,8], with an incidence ranging from 12.5% to 33 % [9,10], and primarily raises the suspicion of idiopathic pulmonary fibrosis in a patient presenting with DPLD, or lung cancer. It is important to keep this in mind, and to start diagnostic approaches as soon as possible, since a timely delay of diagnosis, reported as high as up to 4 months from clinical presentation of digital clubbing to the final diagnosis of lung cancer [11], may have impact on therapy and prognosis.

## Conclusion

If IPF is excluded histologically, clubbing should raise the suspicion of an occult tumour such as non small cell lung cancer [12], which to some extent may be associated with RB-ILD itself [9].

## Abbreviations

D_LCO_: diffusing capacity of the lung for carbon monoxide
HRCT: high-resolution computed tomography
IPF: idiopathic pulmonary fibrosis
PFT: pulmonary function test
RB-ILD: respiratory bronchiolitis-associated interstitial lung disease
TLC: total lung capacity
